# Age, metabolisms, and potential origin of dominant anammox bacteria in the global oxygen-deficient zones

**DOI:** 10.1093/ismeco/ycae060

**Published:** 2024-04-22

**Authors:** Rui Zhao, Irene H Zhang, Amal Jayakumar, Bess B Ward, Andrew R Babbin

**Affiliations:** Department of Earth, Atmospheric and Planetary Sciences, Massachusetts Institute of Technology, Cambridge, MA 02139, United States; Department of Earth, Atmospheric and Planetary Sciences, Massachusetts Institute of Technology, Cambridge, MA 02139, United States; Department of Geosciences, Princeton University, Princeton, NJ 08544, United States; Department of Geosciences, Princeton University, Princeton, NJ 08544, United States; Department of Earth, Atmospheric and Planetary Sciences, Massachusetts Institute of Technology, Cambridge, MA 02139, United States

**Keywords:** anammox, oxygen-deficient zones, nitrogen biogeochemistry, genome evolution

## Abstract

Anammox bacteria inhabiting oxygen-deficient zones (ODZs) are a major functional group mediating fixed nitrogen loss in the global ocean. However, many basic questions regarding the diversity, broad metabolisms, origin, and adaptive mechanisms of ODZ anammox bacteria remain unaddressed. Here we report two novel metagenome-assembled genomes of anammox bacteria affiliated with the *Scalindua* genus, which represent most, if not all, of the anammox bacteria in the global ODZs. Metagenomic read-recruiting and comparison with historical data show that they are ubiquitously present in all three major ODZs. Beyond the core anammox metabolism, both organisms contain cyanase, and the more dominant one encodes a urease, indicating most ODZ anammox bacteria can utilize cyanate and urea in addition to ammonium. Molecular clock analysis suggests that the evolutionary radiation of these bacteria into ODZs occurred no earlier than 310 million years ago, ~1 billion years after the emergence of the earliest modern-type ODZs. Different strains of the ODZ *Scalindua* species are also found in benthic sediments, and the first ODZ *Scalindua* is likely derived from the benthos. Compared to benthic strains of the same clade, ODZ *Scalindua* uniquely encodes genes for urea utilization but has lost genes related to growth arrest, flagellum synthesis, and chemotaxis, presumably for adaptation to thrive in the global ODZ waters. Our findings expand the known metabolisms and evolutionary history of the bacteria controlling the global nitrogen budget.

## Introduction

Anaerobic ammonium oxidation (anammox) is a core process in the global marine nitrogen cycle, estimated to be responsible for a high proportion of fixed nitrogen loss in the pelagic ocean [1–4] and in marine sediments [5, 6]. This process is mediated by microorganisms (i.e. anammox bacteria) in the phylum *Planctomycetota*. Due to inhibition by oxygen [7] in the pelagic ocean, anammox bacteria are restricted to oxygen-deficient zones (ODZs), confined regions with stably limited oxygen that cannot support functional aerobic respiration [8]. The three major ODZs are the Arabian Sea and the Eastern Tropical North and South Pacific (ETNP and ETSP, respectively). First discovered in the Black Sea [9] and Golfo Dulce [10], marine anammox bacteria and their biogeochemical significance have been extensively studied (e.g. [2, 3, 11–13]). Yet basic questions regarding the identity, origin, age, and adaptive mechanisms of anammox bacteria in the ODZ environment remain unaddressed.

Although anammox bacteria are metabolically versatile [14, 15], they need ammonium as an indispensable substrate in the core anammox metabolism, which is scarce across the global ocean. In addition, they can face strong ammonium uptake competition from other organisms, especially the abundant cyanobacteria, which possess higher ammonium affinity than anammox bacteria and are also present in ODZs at certain depth horizons [16]. To possibly overcome this environmental disadvantage, some anammox bacteria are capable of utilizing alternative substrates, such as cyanate and urea [13, 17], two nitrogen compounds commonly present in the ocean [18, 19]. However, without clarity on the overall anammox bacterial diversity in ODZs, extrapolation of these metabolic potentials among anammox bacteria is impossible in this critical habitat.

Anammox bacteria are members of an old functional guild, the last common ancestor of which has been dated to appear 2.32–2.5 billion years ago [20] around the Great Oxidation Event [21]. However, the early-evolved, deep-branching lineage of anammox bacteria (i.e. *Candidatus Bathyanammoxibiaceae*) is mainly found in marine sediments and groundwater but is absent from ODZs [22]. Geochemical proxies from iron speciation and trace metal (e.g. U, V, and Mo) concentrations and isotopic compositions in sedimentary rocks [23] suggest that the earliest emergence time of the modern-type ODZ is ~1.4 Ga in the Mesoproterozoic [24]. Useful approaches to identify the presence of microbial lineages in the geological past, such as isotopic composition and biomarker analyses, have not yet emerged for anammox bacteria because they exhibit similar nitrogen isotope fractionation as denitrifiers [25, 26] and their unique lipid biomarkers (e.g. ladderane [27]) are not known to be preserved for billion-year timescales [28]. Due to the lack of ancient diagnostic signatures, whether anammox bacteria colonized the earliest ODZs and have influenced ocean biogeochemistry ever since remains unclear.

The origin of anammox bacteria in the ODZs is another open question. Anammox bacteria in ODZ waters are mainly free-living [29] and originate either from other ODZs via ocean circulation or from benthic sediments. Considering that a parcel of water takes hundreds of years to connect the geographically separated ODZs, it is unlikely that anammox bacteria of small population sizes can be maintained for such a long time under unfavorable oxic conditions. Instead, it is more conceivable that anammox bacteria in ODZs derive from benthic sediments, especially the coastal sediments that are in direct contact with ODZs. In contrast to the water column, most benthic sediments present an ideal niche for anammox bacteria, as anoxic environments with oxidized (nitrite and/or nitrate) and reduced (ammonium, urea, and cyanate) nitrogen naturally form during early diagenesis [30]. Anammox bacteria in benthic sediments are diverse and affiliated with three different families (i.e. *Ca*. *Scalinduaceae*, *Ca. Bathyanammoxibiaceae*, and *Ca*. *Subterrananammoxibiaceae*) [30–32]. Some of the dominant benthic anammox bacteria are also highly similar to the ODZ *Scalinduaceae* [5]. It is conceivable that benthic sediments may have served as the seed bank of anammox bacteria and have driven the evolutionary radiation of anammox bacteria into the ODZs. However, this hypothesis has not been tested.

To address these questions, we leverage metagenome sequencing data from the global ODZs [33], Arctic marine sediments, and benthic foraminifera to reconstruct high-quality metagenome-assembled genomes (MAGs) of anammox bacteria. Our analyses suggest that there are only two dominant anammox bacteria affiliated with the *Scalindua* genus in the global ODZs, which represent the majority, if not all, of the anammox bacteria in this habitat. We also found their close relatives in marine sediments from different regions. This unique anammox genome dataset from major marine habitats allows us to address questions related to the identities, metabolic functions, origin, emergence time, and adaptive mechanisms of anammox bacteria in the global ODZ environment.

## Materials and methods

### Metagenome sequencing, read recruiting, re-assembly, and manual refinement

We reconstructed MAGs of anammox bacteria from the deep-sequenced metagenome sequencing data of the ODZs of the Arabian Sea and ETNP [33]. All detailed procedures for sample and data collection were described previously in [33]. Briefly, DNA was extracted using the Qiagen AllPrep DNA/RNA Mini Kit following manufacturer protocols. Sequencing was performed at the DOE Joint Genome Institute (JGI) on a NovaSeq (Illumina), resulting in paired-end reads of 151 base pairs in length. After quality control and trimming using Trimmomatic v0.39 [34], the reads were assembled using MEGAHIT v1.2.9 [35]. MAGs were generated from the assembly. The quality-controlled reads were grouped into genome bins using three automatic binners (CONCOCT v1.00 [36], Metabat2 v2.12.1 [37], and Maxbin2 v2.2.6 [38]), implemented in the metaWRAP v1.3 wrapper [39]. All resulting MAGs were classified using GTDB-tk v2.3.0 [40] with the default settings.

To ensure the accuracy of putative anammox bacterial MAGs, we manually checked the genomes by read recruitment, assembly, and re-binning. For each genome, quality-trimmed reads of the sample that showed the highest coverage of this genome were aligned onto the contigs using BBmap [41], and the successfully aligned reads were re-assembled using SPAdes v3.12.0 [42] with the *k*-mers of 21, 33, 55, and 77. After the removal of contigs shorter than 1000 bp, the resulting contigs were visualized and manually re-binned using gbtools v2.6.0 [43]. The input data for visualization and re-binning in gbtools include GC content, taxonomic assignments, and differential coverages of contigs across multiple samples. To generate these input data, the coverages of contigs in each sample were determined by mapping trimmed reads onto the contigs using BBMap v.37.61 [41]. The GC content of individual contigs was also calculated using BBMap v.37.61 [41]. Taxonomic classifications of contigs were assigned by BLASTn [44] according to the taxonomy of the single-copy marker genes in contigs. The quality of the resulting anammox genomes was checked using the “predict” command of CheckM2 v1.0.1 [45] with the default setting. The whole procedure (i.e. reads recruiting, re-assembly, re-binning, and genome quality check) was repeated multiple times until the quality could not be improved further.

### Relative abundance calculation for ODZ *Scalindua* bacteria

To determine the distribution of the two ODZ *Scalindua* in the global ODZs, we leveraged the existing ODZ metagenome sequencing data from the global ODZs: the Arabian Sea [33], ETNP [33, 46, 47], and ETSP [48, 49]. We determined the relative abundances of these two *Scalindua* genomes in a total of 54 metagenome samples in the three major ODZs (Arabian Sea, ETNP, and ETSP) using CoverM using the flags minimap2-sr—min-read-aligned-percent 50—min-read-percent-identity 0.95—min-covered-fraction 0 (https://github.com/wwood/CoverM). For the ETNP and ETSP, the relative abundances of *Scalindua* bacteria and nutrients (nitrite and nitrate) in samples of multiple stations collected during different cruises were plotted by converting the depths to the relative depths at the onset of the ODZ (i.e. the shallowest depth with <3 μM of oxygen). Due to the low genome coverage in some ODZ depths, we also used inStrain [50] with the default settings to estimate the genome coverage breadths as proof of true environmental presence. Only genomes with ~100% breadth are considered to represent genotypes in the samples.

### Genome annotation

All anammox bacterial genomes were annotated using Prokka v1.13 [51], eggnog [52], and BlastKoala [53] using the KEGG database. The functional assignments of genes of interest were confirmed using BLASTp [54] against the NCBI RefSeq database. The metabolic pathways were reconstructed using the KEGG Mapper [55]. Average amino acid identity (AAI) and average nucleotide identity (ANI) between genomes were calculated using EzAAI [56] and FastANI [57] with the default settings, respectively. We used the thresholds proposed in [58] to distinguish different species within the same genus, which share 65%–95% AAI and 95%–98.6% nucleotide identity of the 16S rRNA gene.

### Phylogenetic analysis

To pinpoint the phylogenetic placements of the ODZ *Scalindua* bacteria, we performed phylogenetic analyses for them together with the high-quality genomes of the Planctomycetes phylum included in the GTDB 08-RS214 Release (April 2023). The bacterial 120 single-copy genes were identified, aligned, and concatenated using GTDB-tk v2.3.0 with the “classify_wf” command. The maximum-likelihood phylogenetic tree was inferred based on this alignment using IQ-TREE v1.5.5 [59] with LG + F + R7 the best-fit model selected by ModelFinder [60] and 1000 ultrafast bootstrap iterations using UFBoot2 [61].

A maximum-likelihood phylogenetic tree based on 16S rRNA gene sequences was also used to support the phylogenetic placement of the ODZ *Scalindua* bacteria by adding the sequences and their close relatives to the reference sequences compiled by [32]. Sequences were aligned using MAFFT-LINSi [62], and the maximum-likelihood phylogenetic tree was inferred using IQ-TREE v1.5.5 [59] with GTR + F + R5 as the best-fit substitution model and 1000 ultrafast bootstraps, following the procedure described above.

For the phylogenetic analyses of the key enzymes of anammox bacteria (e.g. cyanase (CynS), urease alpha subunit (UreC), hydrazine synthase alpha subunit (HzsA), and cytochrome *cd1* nitrite reductase (NirS)), the sequences of the two ODZ *Scalindua* were used as the query in the BLASTp [54] search in the NCBI database (>50% similarity and *E*-value of 10^−6^) to identify their close relatives. These sequences were aligned using MAFF-LINSi [62] with reference sequences from [30]. The alignments were then trimmed using trimAl [63] in “automated” mode. Maximum likelihood phylogenetic trees were reconstructed using IQ-TREE v1.5.5 [59] with best-fit evolutionary models and 1000 ultrafast bootstraps.

### Comparative genomic analysis

We performed a comparative analysis of six genomes within *Scalindua* Clade B. Three (*Ca. Scalindua praevalens*, *Ca. Scalindua arabica*, and bin.7) are from ODZs, and the remaining three are from marine sediments. We ran the analysis using Anvi’o v7.1 [64]. All genomes were first annotated using anvi’o against the NCBI’s Clusters of Orthologous Groups (COGs) [65]. The comparative genomic analysis uses BLAST to quantify the similarity between each pair of genes and the Markov cluster algorithm (MCL) [66] (with an inflation parameter of 2) to resolve clusters of homologous genes. The shared and unique genes in the two genomes were identified via functional enrichment analysis [67].

### Molecular clock analysis to date the divergence times of anammox bacteria

To estimate the divergence time of the two ODZ *Scalindua* bacteria from other anammox bacteria, we performed molecular clock analysis using the program MCMCTree from the PAML package (version 4.9j) [68]. This analysis required two input files: (i) a sequence alignment of the PHYLIP format and (ii) a corresponding Newick format phylogenetic tree of the to-be-dated microbial genomes and appropriate reference species. We selected 101 genomes in the analysis, including 37 selected high-quality genomes from the five known anammox bacterial families and 64 reference bacterial genomes. The phylogenetic analysis was based on a concatenation of 26 single-copy genes, which are part of the set of 71 bacterial genes that show little horizontal transfer and therefore are suitable for phylogenetic analysis [69]. After identifying the individual genes in the genomes in Anvi’o v7.1 [64], the sequences of each gene were aligned with MUSCLE [70], and then the individual alignments were concatenated. This alignment in fasta format was converted to the PHYLIP format using Clustal Omega [71] for the subsequent MCMCTree analysis. Also based on the alignment, a maximum-likelihood phylogenetic tree was inferred using IQ-TREE v1.5.5 [59] with the best-fit LG + R6 substitution model. The resulting phylogenetic tree was rooted at desired branches using the tree manipulating tool nwkit [72], and the total sequence number (101) and the tree number (1) were manually added as the top line of the Newick tree file.

With these two input files, the dating analysis was performed in MCMCTree with the approximate likelihood method [73] and the iteration parameters burn-in: 10 000; sampling frequency: 50; number of samplings: 50 000. The analysis was run until convergence. The estimated age ranges of the following four nodes of the tree were manually set in the tree file to run the calibration: (i) the tree root marking the divergence of Cyanobacteria at 3.0 Ga; (ii) the *Nostocales* Crown age of 1.75–2.07 Ga [74]; (iii) the *Aeromonas* Crown age of 0.072–0.479 Ga [75]; and finally the *Vibrio* Crown age of 0.113–0.278 Ga [76].

### Re-analysis of clone library sequencing data of ODZ waters and sediments

To check whether the two MAGs recovered from the Arabian Ocean ODZ can represent ODZs of other locations, we re-analyzed the 16S rRNA gene clone libraries of anammox bacteria in four ODZs: Arabian Sea, off Peru, off Namibia, and Northern Chile. We downloaded the data from the NCBI database using the accession numbers provided [77–79]. A total of seven samples included in these three studies are all from ODZ cores [221 m and 230 m at Station 1 (17.00°N, 68.01°E), 276 m at Station 2 (19.80°N, 64.60°E), and 249 m at Station 23 (15.00°N, 64.00°E) of the Arabian Sea; 52 m at Station 202 (22.64°S, 14.30°E) of the Namibian ODZ; 35 m at Station 4 (12.03°S, 77.49°W) of the Peruvian ODZ; and 50 m at Station Pro2 (20.28°S, 70.28°W) of the northern Chile ODZ]. We aligned all sequences from the four ODZs using MAFFT-LINSi [62] and ran the OTU clustering using the 97% nucleotide identity cutoff, as implemented in Mothur [80]. Representative OTU sequences were added to the backbone 16S rRNA gene sequences and aligned using MAFFT-LINSi [62]. A maximum-likelihood phylogenetic tree was inferred using IQ-TREE [59] with GTR + F + R5 as the best-fit evolutionary model and 1000 ultrafast bootstrap iterations.

Likewise, to understand the anammox community structure in the benthic sediments of the Peru margin, we downloaded the 16S rRNA gene clone library sequences reported in Rich, Arevalo, Chang, Devol, and Ward [81]. The total 82 anammox bacterial sequences were aligned, and OTUs were clustered at the cutoff of 97% nucleotide similarity using Mothur [80]. Representative OTU sequences were also added to the 16S rRNA gene tree by performing phylogenetic analysis using IQ-TREE [59].

We also investigated the presence of the sedimentary relatives of *Ca. Scalindua praevalens* by searching the distribution of GTDB species Scalindua sp022570935 in the Sandpiper database [82], which employs SingleM (https://github.com/wwood/singlem) to search against all public metagenome datasets listed in the NCBI SRA database.

## Results and discussion

### Two novel *Scalindua* anammox genomes from the Arabian Sea ODZ

We used metagenome sequencing data from the global ODZs to recover anammox bacterial genomes. We obtained two MAGs comprising scaffolds that can be distinguished by plotting their scaffold coverages at two Arabian Sea depths (Fig. S1). The two MAGs share an average AAI of only 68.2%, suggesting that they represent two different species [58]. ODZ_A is estimated to be 95% complete with 2.3% redundancy (comprising 40 scaffolds), while ODZ_B is 92% complete with 2.3% redundancy (comprising 87 scaffolds) (Table 1). ODZ_A contains a near-full-length (1569 bp) 16S rRNA gene sequence, whereas ODZ_B does not contain a 16S rRNA gene. These MAGs of high completion and low redundancy levels enable us to perform genome-based analyses with high confidence.

**Table 1:** Summary of anammox bacterial MAGs investigated in this study.

	*Ca. Scalindua variabilis* (ODZ_A)	*Ca. Scalindua praevalens* (ODZ_B)	*Ca. Scalindua communis* (Bin_040)	MT_3L8^b^	MT_3L11^b^	Bsp_12^c^	Cca_14^c^	Vin_18^c^	Bsp_10^c^
Genome size (Mbp)	2.4	1.8	2.7	2.7	2.5	3.0	2.2	4.3	3.1
# Scaffolds	40	87	255	616	270	410	66	711	432
% GC	40.1%	40.3%	39.6%	39.6%	39.2%	38.6	53.1	41.3	39.3
Completion^a^	95.0%	92.3%	91.7%	84.8%	82.1%	83.7%	93.7%	92.0%	86.5%
Redundancy^a^	2.2%	1.5%	1.7%	2.2%	0.8%	5.5%	1.1%	5.7%	8.5%
N50 of contigs	92 585	32 818	18 598	5068	13 613	14 245	51 434	14 900	11 157
# Coding sequences	2318	1712	2677	2602	2358	2540	1999	3327	2655
Coding density	86.7%	88.7%	86.2%	81.6%	80.8%	82.7%	87.9%	82.9%	83.0%
rRNAs	3	3	4	1	3	1	0	0	8
tRNAs	40	41	38	30	31	30	40	39	34

a Estimated by CheckM2.

b MAGs reported by Ref. [108]; the full IDs are B22T3LB and B27T1L11.

c MAGs refined based on data from Ref. [110].

**Figure 1:**
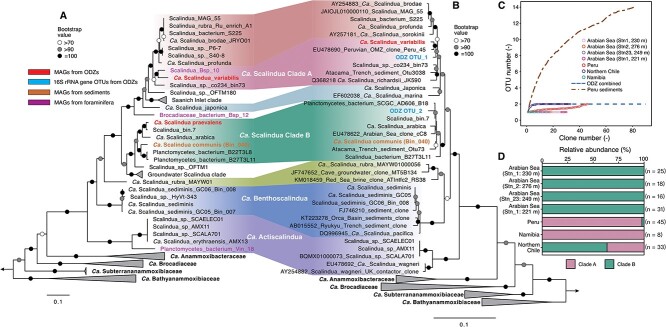
Identities and diversity of anammox bacteria in ODZs. (A) Maximum-likelihood phylogenetic tree of anammox bacteria based on 120 bacterial single-copy genes. (B) Maximum-likelihood phylogenetic tree of anammox bacteria based on the 16S rRNA gene. In both (A) and (B), only sequences in the *Ca. Scalinduaceae* family are shown, while the other four anammox bacteria families are collapsed for readability. Bootstrap values of >70 (*n* = 1000) are shown with symbols listed in the legend. The scale bars show estimated sequence substitutions per residue. The MAGs recovered from the Arabian Sea ODZ and other habitats (i.e., Arctic sediments, foraminifera in Peru margin sediments) are highlighted in different colors as shown in the legend. (C) Low diversity of the *Scalindua* community in global ODZs (including the Arabian Sea, Peru margin, Namibia, and northern Chile), as demonstrated by the rarefaction plot of only ≤2 observed OTUs versus sequenced clones. Also shown are Peru margin sediments and the combination of all ODZs. A total of 176 sequences of the ODZ anammox 16S rRNA gene were analyzed, although the rarefaction curve of the first 90 is shown. (D) Community structure of anammox bacteria in the oxygen-deficient zones (ODZs) in the Arabian Sea, Peru margin, Namibia, and Northern Chile. Clade A and Clade B correspond to* Ca*. Scalindua variabilis and *Ca*. Scalindua praevalens, respectively, as shown in (A) and (B). Panels (C) and (D) are based on a re-analysis of the 16S rRNA gene clone libraries presented in [77–79, 81].

To pinpoint the identities and phylogenetic affiliations of the two ODZ anammox bacterial genomes, we analyzed two sets of phylogenetic markers: (i) 120 bacterial single-copy genes (Fig. 1A) and (ii) the 16S rRNA gene (Fig. 1B). Both analyses suggest that both ODZ MAGs are novel members of the canonical *Scalindua* genus in the *Ca. Scalinduaceae* family. The canonical *Scalindua* genus is notably different from the two deep-branching genera (i.e. g__Scalindua_A and g__SCAELEC01 in GTDB) that are mainly comprised of marine sediment representatives (Fig. 1). The deep-branching sediment members have indeed likely been erroneously named, and we propose two new genus names (i.e. *Ca. Benthoscalindua* and *Ca. Actiscalindua*) to distinguish them from the canonical *Scalindua* genus (Supplementary Text S1).

Both ODZ genomes are divergent enough to represent new *Scalindua* species. They show <90% average nucleotide identities (ANIs) with other known *Scalindua* genomes (Fig. S2), suggesting that these ODZ anammox bacteria cannot be represented by previously known *Scalindua* members from other habitats. Among the previously identified genomes, ODZ_A shows the closest relationship with *Scalindua* sp. co234_bin73 (Fig. 1A), a MAG recovered from anoxic waters of Saanich Inlet [83] and currently the only member of the species *Scalindua* sp018648405 in the GTDB database. The calculated AAI between them is 86.9%, suggesting that *Scalindua* ODZ_A represents a distinct species. Similarly, ODZ_B forms a cluster with several other MAGs from marine environments (Fig. 1A), including SRR1509794_bin.7 (NCBI accession CAJXKT000000000) recovered from the ODZ core of ETNP (125 m depth at 18.9°N 104.5°W) based on existing metagenome sequencing data [47] and *Ca. Scalindua arabica* [84] recovered from the anoxic waters of the Red Sea deep halocline. ODZ_B exhibits a 99.1% AAI with SRR1509794_bin.7 and 91.2% with *Ca. Scalindua arabica*, suggesting that ODZ_B and SRR1509794_bin.7 represent two strains of the same *Scalindua* species.

### Two cosmopolitan *Scalindua* species represent anammox bacterial diversity in ODZs

**Figure 2:**
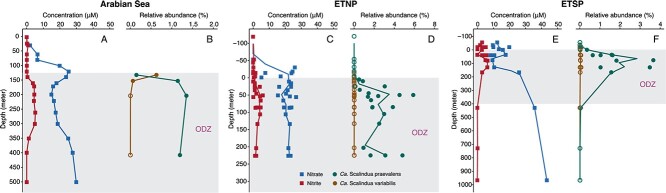
Distribution of the two *Scalindua* MAGs in three major ODZs. Profiles and nitrate and nitrite, and relative abundances of the two *Scalindua* bacteria in the Arabian Sea (A, B), eastern tropical North Pacific (ETNP) (C, D), and eastern tropical South Pacific (ETSP) (E, F). The relative abundances of the two ODZ anammox bacteria are determined by mapping multiple sets of metagenome sequencing reads onto the two *Scalindua* bacterial genomes. To integrate samples of different stations into a coherent profile for ETNP and ETSP, actual depths are converted to depths relative to the onset of the ODZ core. The circles represent individual samples, while the lines denote moving means. The absence of the *Scalindua* bacteria in the analyzed metagenomes (i.e. with <1× coverage or ≪99% genome mapping breadth) is marked by open circles. The waters within the ODZs are marked by a gray box in each area.

Although sourced from the Arabian Sea metagenome sequencing dataset, we determined whether the two *Scalindua* MAGs exist in other major ODZs by recruiting reads from the existing metagenome sequencing data [33]. Competitive mapping [50] against all available *Scalindua* genomes simultaneously reveals that only the ODZ-derived genomes (ODZ_A, ODZ_B, and *Scalindua* bin.7) can represent genotypes in the ODZs and that other *Scalindua* genomes are not truly present in the ODZs (due to either the <1× coverage or ≪99% coverage breadth), even if some others can recruit small fractions of reads (Supplementary Data S1). The highest relative abundance of *Scalindua* we find is 6% of the total community in the ETNP ODZ (Fig. 2). While seemingly up to 6% may indicate anammox bacteria are not important, they routinely represent a small fraction of the total communities [85, 86], even though they drive a considerable fraction of fixed nitrogen loss [1, 2, 87]. The two *Scalindua* bacteria are confined within the ODZ cores and not detectable in oxygenated waters above or below the ODZs (Fig. 2), consistent with the premise that oxygen inhibits the anammox metabolism and therefore the growth of these specialized organisms [7, 88]. The occurrence of anammox bacteria overlaps with the accumulation of nitrite (Fig. 2), suggesting this resource remains readily available. While ODZ_B is present in all analyzed metagenomes, ODZ_A is only present in the 130 m and 150 m metagenomes of the Arabian Sea ODZ (Fig. 2B), suggesting that ODZ_B is the more prevalent anammox bacterium while ODZ_A is variable. We provisionally name ODZ_B *Candidatus Scalindua praevalens* (prevalent in English) and ODZ_A *Candidatus Scalindua variabilis* (variable in English), to highlight their contrasting abundances in the ODZs (see Etymology description).

Anammox bacterial communities in ODZs have been characterized by extremely low diversity (i.e. two clusters within the *Scalindua* genus) based on PCR studies (e.g. [77]). However, given the potential PCR primer biases [89] and the recently discovered anammox families (e.g. *Ca. Bathyanammoxibiaceae* [22], *Ca. Subterrananammoxibiaceae* [32], and *Ca. Anammoxibacteraceae* [90]), the limited diversity implied by PCR analysis should be verified by more extensive genomic analysis. We assess it using the deep-sequenced, primer bias-free metagenome data from only the Arabian Sea and ETNP because deeply sequenced metagenome data are lacking from ETSP. In either individual metagenome assemblies or co-assemblies of these two ODZs, there is maximally only one Brocadiales 16S rRNA gene sequence, which matches either *Ca. Scalindua variabilis* or *Ca. Scalindua praevalens*, but not any anammox bacteria outside the *Scalinduaceae* family (Table S1). Re-analyzing previous 16S rRNA gene clone library sequencing data from the Arabian Sea, Peru margin, northern Chile, and Namibia [77–79], we find our new *Scalindua* MAGs encompass the entire diversity of the anammox community across all these sites. The total 176 anammox 16S sequences recovered from the four ODZ locations clustered into only two OTUs (operational taxonomic units, 97% nucleotide identity cutoff) (Fig. 1C). Anammox bacteria in all ODZ samples are composed exclusively of either OTU_1 or OTU_2, except that both OTUs are detected in the Peru and northern Chile ODZs (Fig. 1C and D). Clone library sequencing of a single sample of the Peruvian ODZ (Station 4, 12.03°S, 77.49°W, 35 m depth with 6 μM oxygen, [77]) suggests that the anammox community was dominated by Clade A, which is different from our assessment based on shotgun metagenome sequencing and might be caused by the differences of samples used or spatiotemporal changes of the local anammox bacterial community. Our metagenome analysis independently validates the previous finding that ODZ anammox bacteria are of low diversity [77, 85]. This low ODZ diversity stands in stark contrast to the high diversity of anammox bacteria in benthic sediments (e.g. 14 OTUs are recovered via the same method from sediments underlying the Peru ODZ; Fig. 1C).

The 16S rRNA gene sequences of the two clone-library OTUs match well with the *Scalindua* MAGs recovered from ODZs. Although the *Ca. Scalindua praevalens* MAG does not contain a 16S rRNA gene; it shows 97.6% AAI with SRR1509794_bin.7, suggesting that these two MAGs represent an identical species and are interchangeable. The match between OTU_1 and *Ca. Scalindua variabilis* is evidenced by the 16S rRNA gene identity of 98.8% over 1526 base pairs between them. The same is true between OTU_2 and SRR1509794_bin.7 (i.e. *Ca. Scalindua praevalens*), as they show 99.1% 16S rRNA gene similarity. The matches between the OTUs and ODZ *Scalindua* MAGs are supported by including the two OTUs in the 16S rRNA gene phylogenetic tree of anammox bacteria (Fig. 1B). Pair-wise 16S rRNA gene identity calculation suggests that the divergences between *Scalindua* members are not as significant as shown by the ANIs, as exemplified by the >98.5% 16S rRNA gene identifies between different species in *Scalindua* Clade B (Fig. S3). This discrepancy may indicate that the 16S rRNA gene has a lower evolution rate than other genes in the *Scalindua* genomes. Nevertheless, the two *Scalindua* MAGs from ODZs show the highest identities to the two 16S rRNA gene OTUs clustered from the available ODZ clone libraries. Thus, the two *Scalindua* MAGs recovered in this study represent the most dominant, if not all, anammox bacteria diversity in the global ODZs.

### Metabolic potential of the two dominant *Scalindua* in ODZs

**Figure 3:**
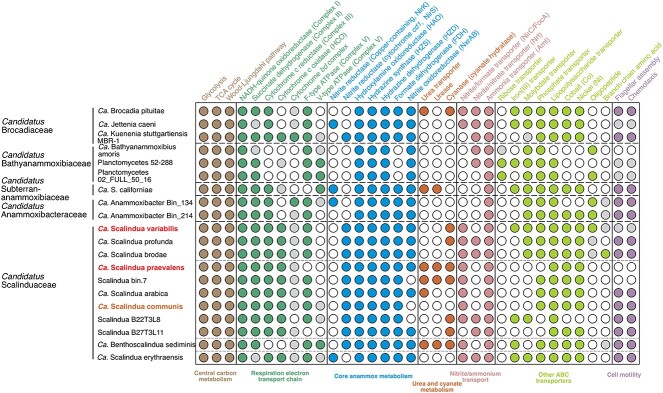
Metabolic potential is encoded in anammox bacteria genomes. Filled color circles indicate the presence of full pathways, open circles indicate the absence, and light gray circles indicate the presence of partial pathways. Genomes recovered from ODZs and AMOR sediments are highlighted. Also shown are high-quality genomes from the other four anammox bacterial families for comparison. The family-level affiliations of the anammox bacteria are indicated on the left-hand side.

Similar to other characterized anammox bacteria, the two ODZ *Scalindua* have all known essential genes for the core anammox metabolism. In particular, they contain the diagnostic hydrazine synthase (HZS) [91, 92] for combining ammonia and nitric oxide (NO) to generate hydrazine (Fig. 3). The functional gene tree of the HZS alpha subunit (HzsA) exhibits a similar topology to the phylogeny (Fig. 1A and B), confirming the lineage-specific HZS in the two ODZ *Scalindua* bacteria (Fig. S4). Although multiple copies of HZS have been observed in some anammox bacteria genomes (e.g. *Ca. Kuenenia stuttgartiensis* [14] and *Ca. Benthoscalindua sediminis* [30]), there is only one copy of HZS in both ODZ *Scalindua* genomes. Like other marine anammox bacteria [93], the beta and gamma subunits of HZS (hzsB and hzsC) are fused into a single protein, typically annotated simply as hzsB. The ODZ *Scalindua* genomes also contain the unique anammox hydrazine dehydrogenase (HAO) [94] that can oxidize hydrazine to N_2_ (Fig. 3). Like other *Scalindua* bacteria, they encode a nitrite transporter (NirC, homologous to the formate transporter FocA) to assimilate nitrite (Fig. 3). For the generation of NO, the direct precursor of hydrazine [91, 92], they can use cytochrome *cd1*-containing nitrite reductase (NirS) to reduce nitrite to NO (Fig. 3). The tree of anammox bacterial NirS (Fig. S5) shows a congruent topology with trees based on other conservative phylogenetic markers (Fig. 1), indicating that NirS is an essential trait of members of the *Ca. Scalinduaceae* family. In addition, they also contain nitrite oxidoreductase (NXR) that can provide electrons for carbon fixation through the Wood–Ljungdahl pathway (Fig. 3). Transporters for oligopeptides and branch-chain amino acids are encoded in members of the *Ca. Bathyanammoxibiaceae* and *Ca. Anammoxibacteraceae* members, and some members of Clade A of the *Scalindua* genus, but not any members of Clade B (Fig. 3). The absence of ATPase in *Ca. Scalindua praevalens* (Fig. 3) may result from incomplete genome reconstruction or assembly errors.

**Figure 4:**
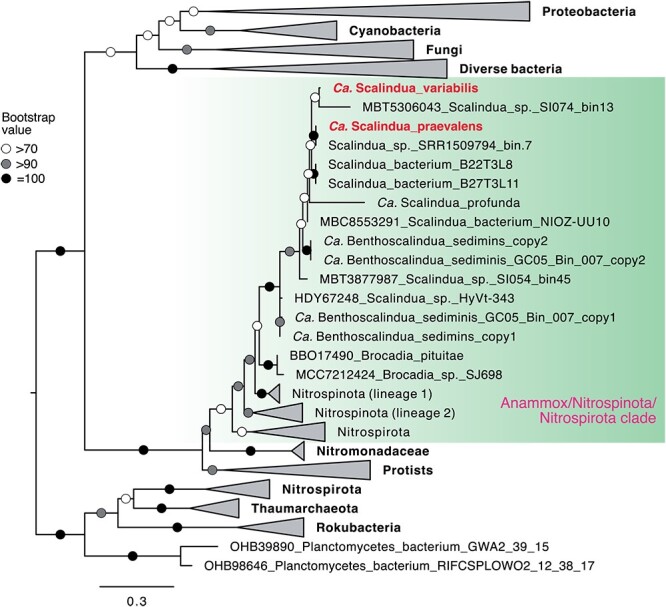
Maximum-likelihood phylogenetic tree of cyanase in anammox bacteria. For simplicity, only sequences of the marine anammox/Nitrospinota/Nitrospirota clade are shown, while other clades are collapsed. The two ODZ anammox MAGs are noted in bold. Bootstrap values of >70 (*n* = 1000) are shown with symbols listed in the legend. The scale bar shows the estimated sequence substitutions per residue.

These high-quality *Scalindua* genomes reconstructed directly from ODZ samples provide support for their widespread capacity for utilizing diverse reduced nitrogen substrates. Both ODZ *Scalindua* bacterial genomes contain a cyanase (Fig. 3), an enzyme that can degrade cyanate to ammonium and CO_2_ [95]. Given that these two *Scalindua* represent all of the known ODZ anammox bacteria diversity (Fig. 1), the presence of cyanase in both ODZ *Scalindua* bacteria indicates this metabolic trait is ubiquitous among ODZ anammox bacteria and provides direct support to the observation that cyanate stimulates anammox reaction rates [13], presumably by providing a source of ammonium. Furthermore, this observation confirms that ODZ *Scalindua* can degrade cyanate directly. All but two cyanase-containing anammox bacteria are members of the marine *Ca. Scalinduaceae* family (either anoxic seawater or sediments) (Fig. 4). Cyanate in pelagic ODZs is produced by organic matter degradation and phytoplankton release [96] and maintained at low concentrations due to highly active uptake relative to urea and ammonium in ODZs [18, 19]. The active expression of anammox cyanase genes [17, 93] suggests their role in consuming available cyanate.

Except for *Ca. Benthoscalindua sediminis*, which contains two cyanase variants [30], and all other anammox bacteria contain only one (Fig. 4). Phylogenetically, the cyanase sequences of anammox bacteria form a monophyletic clade that clusters with nitrite-oxidizing bacteria from Nitrospirota and Nitrospinota, two phyla distinct from the anammox-harboring *Planctomycetota*. This indicates that cyanases in anammox bacteria and nitrite-oxidizing bacteria likely result from horizontal gene transfer (HGT) events (Fig. 4), although the timing and directions of such transfers necessitate further reliable dating of these lineages, especially the nitrite-oxidizing phyla. Phylogenetic similarity (inferring HGT) between anammox bacteria and nitrite-oxidizing Nitrospirota and Nitrospinota has been observed for NXR [32, 97, 98]. Cyanase is potentially another functional apparatus that has been transferred between these two important nitrogen-cycling groups and may partly govern the ecological interactions between them.

Urea is also an important substrate for nitrogen-cycling organisms in the ocean [17, 99], which mainly originates from the metabolic activities of marine organisms, such as zooplankton excretion [100] and cellular decomposition [101]. *Ca. Scalindua praevalens* contains both a urease and a urea transporter (Fig. 3). Given the abundance of this organism across ODZs (Fig. 2), it is likely that many ODZ anammox bacteria can assimilate and hydrolyze urea and produce intracellular ammonium. This observation provides strong support for the previous observation that urea can enhance anammox rates in the ETSP ODZ [13]. Urea concentrations of up to 2 μM have been detected in both oxic and anoxic seawater [19, 84], while concentrations of ammonium in ODZ waters are only on the order of tens to hundreds of nanomolars [13, 102], indicating that urea can be important for ODZ anammox bacteria when ammonium is limiting. In contrast to cyanate, urease is not widespread in anammox bacteria or the *Ca. Scalinduaceae* family (Fig. 3). Urease-containing anammox bacteria are only found in *Ca. Scalindua praevalens*, several planktonic *Scalindua* genomes from Saanich Inlet [83], *Ca. Benthoscalindua sediminis* from marine sediments, and *Ca. Subterrananammoxibius californiae* [32] (Fig. 3). Phylogenetic analysis of anammox urease sequences reveals that they form a clade separate from other nitrogen-cycling groups (Fig. S6), and thus urease may have a different evolutionary route than cyanase and NXR among anammox bacteria.

### Radiation of *Scalindua* into ODZs between the carboniferous and cretaceous

**Figure 5:**
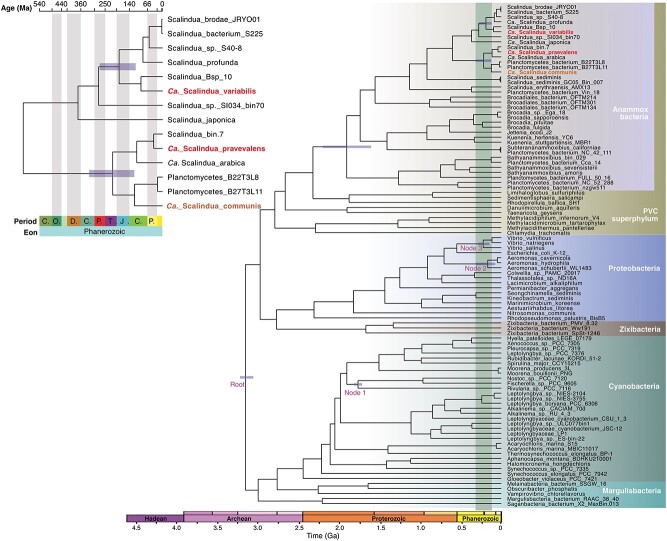
Molecular clock dating the divergence times of the two ODZ *Scalindua* bacteria. The depicted chronogram is a time-calibrated species tree that was initially generated by a concatenated alignment of 26 single-copy bacterial genes. The tree root and three nodes for calibrations (Node 1, 2, 3) are labeled in purple. Posterior distributions were generated by sampling the key Markov Chain Monte Carlo analysis every 1000 generations, with a 25% burn-in. Blue horizontal bars denote 95% confidence intervals. For simplicity, only the bars for the nodes within the anammox lineage discussed in the main text are shown. The inset highlights the divergence times of the two ODZ *Scalindua* bacteria within the Phanerozoic eon.

To assess for how long the two ODZ *Scalindua* bacteria have influenced ocean chemistry over geological history, we performed a molecular clock analysis to estimate the earliest divergence time of their ancestors. Although the appearance time of the last common ancestors of all anammox bacteria was previously dated to be 2.32–2.50 Ga [21], the evolutionary history of *Ca. Scalinduaceae* members, especially the subset of ODZs, have not yet been constrained. Our analysis suggests that the origins of the clades containing the two ODZ *Scalindua* bacteria broadly fall into the Phanerozoic eon (<540 Ma, Fig. 5). In particular, the origin of the crown group of the clade containing *Ca. Scalindua praevalens* is dated to 210 Ma [95% highest posterior density (HPD) interval, 120–310 Ma], while that of *Ca. Scalindua variabilis* is dated to 190 Ma (95% HPD interval, 110–270 Ma) (Fig. 5). The origins of these two ODZ *Scalindua* bacteria are constrained in the range between the Carboniferous and Cretaceous (Fig. 5), a time when atmospheric oxygen levels were fluctuating but close to the present level [103] and the deep ocean was likely already oxygenated [104]. Considering that the earliest modern-type ODZ structure can be traced much earlier to the Mesoproterozoic (~1.4 Ga [24]), the two *Scalindua* bacteria dominating the modern ODZs are much younger. Despite the known limitations of molecular clock analyses (e.g. relatively large uncertainty and assumptions), our results suggest that the appearance of the two dominant anammox bacteria in the ancient oceans occurred at least 1 billion years later than the emergence of their ideal niche (i.e. minimal oxygen but sufficient supply of both oxidized and reduced nitrogen compounds) in the ocean. Our divergence time constraints raise the question of whether anammox bacteria were present and played a critical role in nitrogen cycling in the first billion years after the emergence of ODZ structure (i.e. 1.4–0.3 Ga). If yes, their niche was filled by extinct, or currently very rare, organisms. Otherwise, the ODZ nitrogen consumption during this period was likely mediated by only denitrifiers and ammonium accumulated. Nevertheless, our molecular clock analysis results suggest that the two modern *Scalindua* bacteria have existed in the ODZs and therefore have influenced global biogeochemical cycles for <310 million years.

### Sedimentary origin of the ODZ *Scalindua* bacteria in geological history

**Figure 6:**
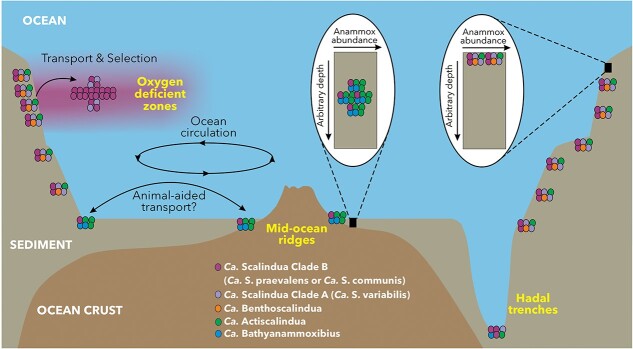
Anammox bacteria diversity across the marine environment. Anammox bacteria species are represented by different colors (not quantitative). ODZs anammox bacteria likely derive from benthic sediments with selection by local conditions. Anammox bacteria are widespread in sediments on the continental shelf, at mid-ocean ridges, and within hadal trenches. Because the overlying water columns in the mid-ocean ridge and hadal trenches contain no anoxic waters, the anammox bacteria found there must be transported by seawater circulation, which may be facilitated by the protection from animals, such as foraminifera. In sediments, diverse anammox bacteria dominate their ideal subsurface niche, the subsurface nitrate-ammonium transition zone, the depth of which depends on organic matter supply and oxygen penetration.

The redox conditions in the ocean have changed dramatically in the geological history of Earth (e.g. [105]). Most of these redox conditions may not be habitable for anammox bacteria due to a lack of essential substrates or desired redox conditions. Before the radiation of *Scalindua* bacteria into the ocean, anammox bacteria (preferentially living in modern marine sediments and groundwater) existed for ~2.3 billion years [21], aligning with the Great Oxidation Event (Fig. 5). When the ocean became habitable for anammox bacteria, the first cells occupying the anammox niche may have diversified from other bacteria or have been transported from other habitats. We hypothesize that the first cells of the *Scalindua* bacterial lineages currently residing in modern ODZ waters ultimately originated from the benthos in geological history because the benthos harbor anammox bacteria of higher diversity (containing three families) [22, 30–32] and longer evolutionary history (Fig. 6). Considering that the deep ocean was oxygenated (since ~420 million years ago [104]) around the emergence of the two ODZ anammox bacteria, the radiation of anammox bacteria into the ocean most likely occurred in coastal benthic sediments that intersect with ODZs, where lateral transport of metals like Fe and Mn to ODZs has been previously documented by geochemical analyses (e.g. [106, 107]).

We tested this hypothesis at the Peru margin, where the ODZ is in direct contact with sediments [106]. We re-analyzed the 16S rRNA gene clone library from seven sediment stations on the Peru margin [81]. The total 82 sequences clustered into 14 OTUs (97% nucleotide identity cutoff), indicating that the benthic sediments exhibit a much higher anammox bacterial diversity than the ODZs off Peru (Fig. 1C). Although the majority of the sediment sequences are affiliated with the genus *Scalindua*, especially Clade A, they also contain some sequences from the genera *Ca. Benthoscalindua* and *Ca. Actiscalindua* (Fig. S7). Importantly, however, the two most dominant sediment OTUs share >98.6% 16S rRNA gene identities with the two ODZ *Scalindua* MAGs (Table S2), suggesting that the two major ODZ *Scalindua* bacteria are also dominantly present in the local benthic sediments of the Peru margin. The same *Scalindua* species present in benthic sediments and the overlying ODZ waters off the Peru Margin reflect potential microbial exchange between the two major habitats of marine anammox bacteria, but less diversity in the ODZs may arise from natural selection winnowing the community. Although we propose that the sediment-to-water transition is the ultimate geological origin of the *Scalindua* bacteria in ODZs, the exact origin of every *Scalindua* cell currently living in the global ODZ waters may be diverse and cannot be resolved by our data.

### Relatives of *Ca. Scalindua praevalens* are widespread in marine sediments

To check if the Peru margin sediments are the only location of the sedimentary relatives of *Ca. Scalindua praevalens*, we generated metagenome sequencing data from multiple marine sediment locations. We obtained a high-quality MAG (Bin_040) (91.4% complete with 1.6% redundancy) and also a 16S rRNA gene (Table 1) from Arctic Mid-Ocean Ridge sediments. This MAG has two close relatives (B22T3LB and B27T1L11, classified as *Scalindua* sp022570935 in GTDB) of lower completeness levels (<85%, Table 1) that were recovered from Mariana Trench sediments [108] (Fig. 1A and B). While Bin_040 is not abundant (<0.16% of the total community) in the Arctic Mid-Ocean Ridge sediments where it originates, it represents the dominant anammox bacterium in the Atacama Trench sediments [5, 109] (Supplementary Text S2). Bin_040, together with the two Mariana Trench MAGs, form a lineage highly related to that of *Ca. Scalindua praevalens* (Fig. 1A and B). When searching in the sandpiper database [82], members of this genus are present with >0.01% relative abundances in 36 globally distributed marine sediment samples (Table S3). They are present in sediments not only beneath ODZs (ETNP and Arabian Sea), but also in areas without ODZs (e.g. West Pacific, South Atlantic Ridge, South China Sea, and Nordic Seas). We provisionally name Bin_040 *Ca. Scalindua communis* to reflect its presence across a wide range of marine sediments. Therefore, close relatives of *Ca. Scalindua praevalens* inhibit benthic sediments in widespread locations (Fig. 6). This distribution implies continuity of the benthic assemblages so that the exchange of *Ca. Scalindua praevalens* between the water column and benthic sediments can potentially happen globally, but the organism can only proliferate in favorable ODZ waters.

Novel transport mechanisms are perhaps required for the spread of anammox bacteria in global marine sediments. Considering that much of the global ocean is well-oxygenated, anammox bacteria have to be shielded from inhibitory oxygen during transport. Given that anammox bacteria are preferentially free-living [29] and not enriched on particles [49], they may use other objects as rafts to spread. Symbiosis is one possible mechanism to resist natural inhibition by gaining protection from another organism. In support of this, we found four MAGs of anammox bacteria (Table 1) in association with foraminifera in the Peru margin sediments [110]. These foraminifera-associated anammox bacteria exhibit high diversity and are affiliated with three genera in two different families (*Ca. Scalinduaceae* and *Ca. Bathyanammoxibiaceae*) (Fig. 1A). In particular, Bsp_10 is closely related to *Ca. Scalindua variabilis* (Fig. 1A), suggesting that foraminifera may have served to shuttle this particular species between sediments and the ODZ (Fig. 6). Considering that the crown group of foraminifera has been dated to appear in the Neoproterozoic (690–1150 Ma [111]), they were likely available as rafts during the radiation of the two ODZ *Scalindua* bacteria from benthic sediments to the water column. More work on the phylogenetic range of anammox-containing foraminifera in diverse locations is required to confirm this provocative hypothesis.

### Genes unique to the most dominant ODZ *Scalindua*

Given that different strains of the same species are present in the ODZs and marine sediments, we compared their genomes to identify any genes that may result in this apparent habitat differentiation among marine anammox bacteria. We focus on the clade harboring *Ca. Scalindua praevalens* (Clade_B) because it contains three highly related representatives of oxygen-deficient waters (*Ca. Scalindua praevalens*, bin.7, and *Ca. Scalindua arabica*) and also three representatives of sedimentary origin (*Ca. Scalindua communis*, B22T3LB, and B27T1L11), while such data are not available for the clade harboring *Ca. Scalindua variabilis* (Clade A). In the *Ca. Scalinduaceae* family, the four cultured members are all from coastal sediments (*Ca. Scalindua brodae*, *Ca. Scalindua profunda*, *Ca. Scalindua japonica*, and *Ca. Scalindua erythraensis*) and tend to have larger genome sizes than the uncultured ones (4.7 ± 0.3 Mbp for cultured vs. 3.8 ± 1.1 Mbp for uncultured) (Fig. S8, Welch’s test, *P* = 0.0025), which could be attributed to the possibility that the former have more metabolic potential and less dependences on other community members and thus may be easier to culture in the laboratory. Despite smaller genome sizes (1.8–2.1 Mbp) than their sediment counterparts (2.6–2.8 Mbp), the ODZ *Scaliundua* genomes have some unique genes, including those encoded for urease. Urease is present in two of the three ODZ genomes but not in sediments, which may enable them to exploit urea in seawater as an alternative substrate. In addition, we found that the ODZ genomes lack methylmalonyl-CoA mutase, which is present in the three sediment genomes. Methylmalonyl-CoA mutase is one of the most abundant cobamide-dependent enzymes in bacterial genomes [112] and can impair cobalamin acquisition and arrest growth [113], which is advantageous for sedimentary microbes under extreme energy limitation [114]. Notably, the ODZ genomes also have no genes for flagellar synthesis and chemotaxis, which, if expressed, would otherwise enhance microbial colonization of particles [115]. However, anammox bacteria in ODZs are free-living [29], autotrophic, and their substrates are mainly dissolved in seawater. Avoiding attachment to particles can help them stay within their preferred niche rather than being exported deeper. It is worth noting that flagella synthesis and rotation are highly energy-consuming [116], and flagella themselves have not been observed on anammox bacteria under transmission electron microscopy (except for *Ca. Scalindua profunda*). Similarly, chemotaxis has never been demonstrated in cultured anammox bacteria. Whether flagella synthesis and chemotaxis are functional in non-ODZ anammox bacteria remains unclear. Nevertheless, these genomic changes (gene gain for urea utilization and gene loss for flagella synthesis and chemotaxis) may have played important roles in streamlining their genomes and enabling them to adapt to planktonic lifestyles in global modern ODZs. Other adaptive mechanisms on the cellular level (e.g. substrate affinities, inhibition constants, and growth kinetics) warrant further laboratory characterization of marine anammox bacteria, although cultivation currently escapes conventional methods. Transcriptome and proteome analyses of water samples from more ODZs will be useful to confirm the described functional potential and their ubiquity in the global ODZs.

## Conclusion

We documented two novel *Scalindua* bacteria that represent most, if not all, anammox bacteria in the global ODZs. The genome contents and the relative abundances of these two bacteria indicate that all ODZ anammox bacteria can utilize cyanate and many can also use urea as reduced nitrogen substrates, underscoring the importance of these compounds as a source of ammonium and a new link in global ocean nitrogen cycling. These anammox bacterial communities maintain low diversity but high similarity between geographically separated ODZs and likely stem from benthic sediments where their close relatives have been found. The evolutionary radiation of these *Scalindua* bacteria from sediments into the ODZs is estimated to have happened less than ~310 million years ago, ~1 billion years later than the earliest appearance of the chemical conditions identifiable as modern-day ODZs. The appearance of anammox bacteria in the ODZs may have resulted from their sediment-to-ODZ transport, before which either ammonium accumulated or this niche was filled by other organisms. Compared to their sedimentary counterparts, the ODZ *Scalindua* bacteria acquired urease but also lost genes involved in growth arrest, flagellar synthesis, and chemotaxis. Our findings shed new light on the ecology and history of one of the major functional guilds that govern nitrogen availability in the global ocean.

### Etymology description

#### Taxonomic consideration of “*Ca. Scalindua praevalens*”

A novel *Scalindua* bacterium represented by a genome reconstructed from metagenome data of the Arabian Sea. Prae.va’lens. L. part. Adj. *praevalens*, very powerful. This bacterium represents the majority of anammox bacteria inhabiting the ODZs of the Arabian Sea, ETNP, and ETSP. The dominant anammox bacterial phylotype in the Arabian Sea was previously named *Ca. Scalindua arabica* [77]. However, *Ca. Scalindua arabica*, as a genomic species, has been used to name a MAG retrieved from the Red Sea deep halocline [84], which is different (91% AAI) from the dominant MAG directly recovered from ODZs. Because of this and also the ubiquity of this ODZ bacterium, we choose to provisionally name it *Ca. Scalindua pravaelens*.

#### Taxonomic consideration of “*Ca. Scalindua variabilis*”

A novel *Scalindua* bacterium represented by a genome reconstructed from metagenome data of the Arabian Sea. va.ri.a’bi.lis. L. fem. Adj. variabilis, variable, changeable. This bacterium represents the dominant anammox bacteria in the upper part of the Arabian Sea ODZ and also some depths in the ETSP, but could be rare in other ODZ depths and locations. It represents the ODZ anammox bacterial phylotype (OTU_1 in Fig. 1B), previously thought to be affiliated with the “Scalindua sorokinii/brodae clade” [77]. However, because *Ca. Scalindua brodae* and *Ca. Scalindua sorokinii* (i) share <98% 16S rRNA gene identity with OTU_1 and (ii) fall into a different phylogenetic clade from OTU_1 (Fig. 1A and B), these two species do not appropriately represent OTU_1 derived from ODZs. Therefore, we propose that *Ca. Scalindua variabilis* directly recovered from ODZs should represent an ODZ anammox bacteria species.

#### Taxonomic consideration of “*Ca. Scalindua communis*”

A novel *Scalindua* bacterium represented by a genome reconstructed from metagenome data of Arctic Mid-Ocean Ridge sediments but is present in many other locations. Com.mu’nis. L. fem. Adj. communis, common. It is the sediment relative of *Ca. Scalindua praevalens* and has genomic relatives from Mariana Trench sediments.

## Supplementary Material

ODZ_anammox_ISMEComm_SI_ycae060

Table_S3_Distribution_of_Scalindua_sp022570935_ycae060

Supplementary_Data_S1_inStrain_analysis_ycae060

## Data Availability

All raw sequencing data used in this study are available in the Joint Genome Institute (JGI) data portal associated with the naming convention AMALJGI-DNA-#, while metagenome assemblies and initial MAGs are available in the NCBI Short Reads Archive under the Bioproject number PRJNA955304. The two ODZ *Scalindua* genomes are available under the Bioproject number PRJNA1023948 with accession numbers JAWKDT000000000 (*Ca. Scalindua variabilis*) and JAWKDU000000000 (*Ca. Scalindua praevalens*).
