# Supplementary material for: Age, metabolisms, and potential origin of dominant anammox bacteria in the global oxygen-deficient zones
Source: ISME Commun. 2024 Apr 22;4(1):ycae060. doi: 10.1093/ismeco/ycae060 (PMC11104535; doi:10.1093/ismeco/ycae060)
Supplement: ODZ_anammox_ISMEComm_SI_ycae060 [file odz_anammox_ismecomm_si_ycae060.docx]

**Supplementary Information**

**Supplementary Text 1**

***Revisiting the classification and naming of anammox bacteria within the* Ca. *Scalinduaceae family***

Phylogenetic analyses indicate that in addition to *Ca.* Scalindua, there are two new genera within the *Ca*. Scalinduaceae family. The first is composed of the strains originally called *Ca.* Scalindua sediminis mainly recovered from marine sediments of the Arctic Mid-Ocean Ridge [1] and related cloned sequences from other sediment locations (Fig. 1B). We propose to rename it *Ca.* Benthoscalindua (corresponding to g__Scalindua_A in GTDB) to distinguish it from the true *Scalindua* genus. The second falls at the basal branch of the *Ca.* Scalinduaceae family and is composed of *Ca.* Scalindua erythraensis originally enriched from coastal sediments [2] and related strains [3]. We propose to name it *Ca.* Actiscalindua (corresponding to g__SCAELEC01 in GTDB).

**Supplementary Text 2**

***The dominance of* Ca. *Scalindua communis in Atacama Trench sediments***

We determined whether *Ca.* Scalindua communis (Bin_040) recovered from Arctic sediments is also present in the hadal sediments beneath the Atacama Trench [4]. The similarity of geochemical stratification and microbial communities between sediments of these two locations has been shown previously [5]. Based on the re-analysis of the 16S rRNA gene amplicon sequencing data of nine Atacama Trench cores [5], there are two OTUs (OTU_73 and OTU_3038) dominant in the anammox bacterial community. Across the total 152 sediment samples, OTU_73 comprises 73.8% of the anammox bacterial community while OTU_3038 accounts for 7.8%. The 16S rRNA gene of *Ca.* S. communis shows a 100% identity to OTU_73 (Fig. 1B), suggesting that it can represent the most abundant *Scalindua* bacteria in the Atacama Trench sediments. This is consistent with the previous description that anammox bacteria in Atacama Trench sediments are most similar to some *Scalindua* bacteria inhabiting the Arabian Sea oxygen deficient zone [6].

**Supplementary Figures**


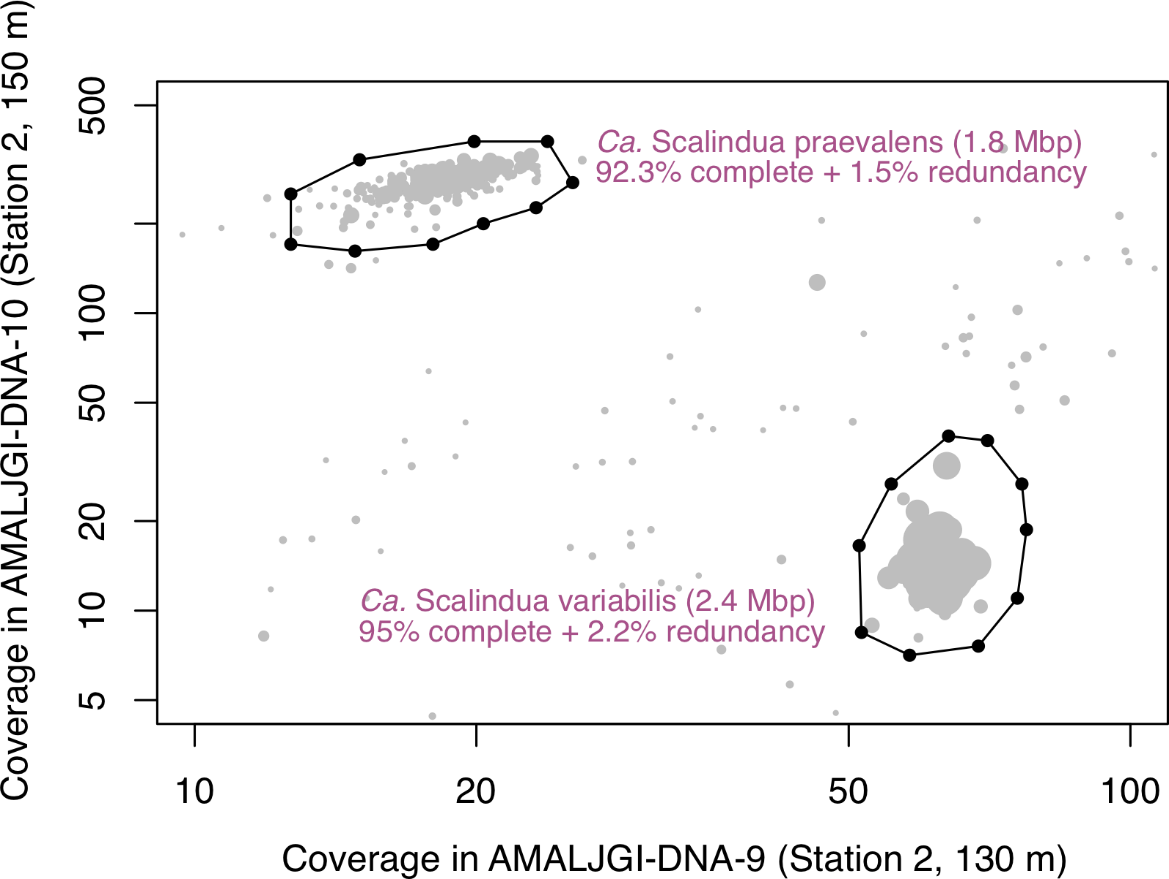


**Fig. S1. Differential coverages of the two *Scalindua* MAGs in two depths (130 m and 150 m) within the Arabian Sea oxygen deficient zone.** The scaffolds belonging to the putative genomes are enclosed by two manually defined decagons.


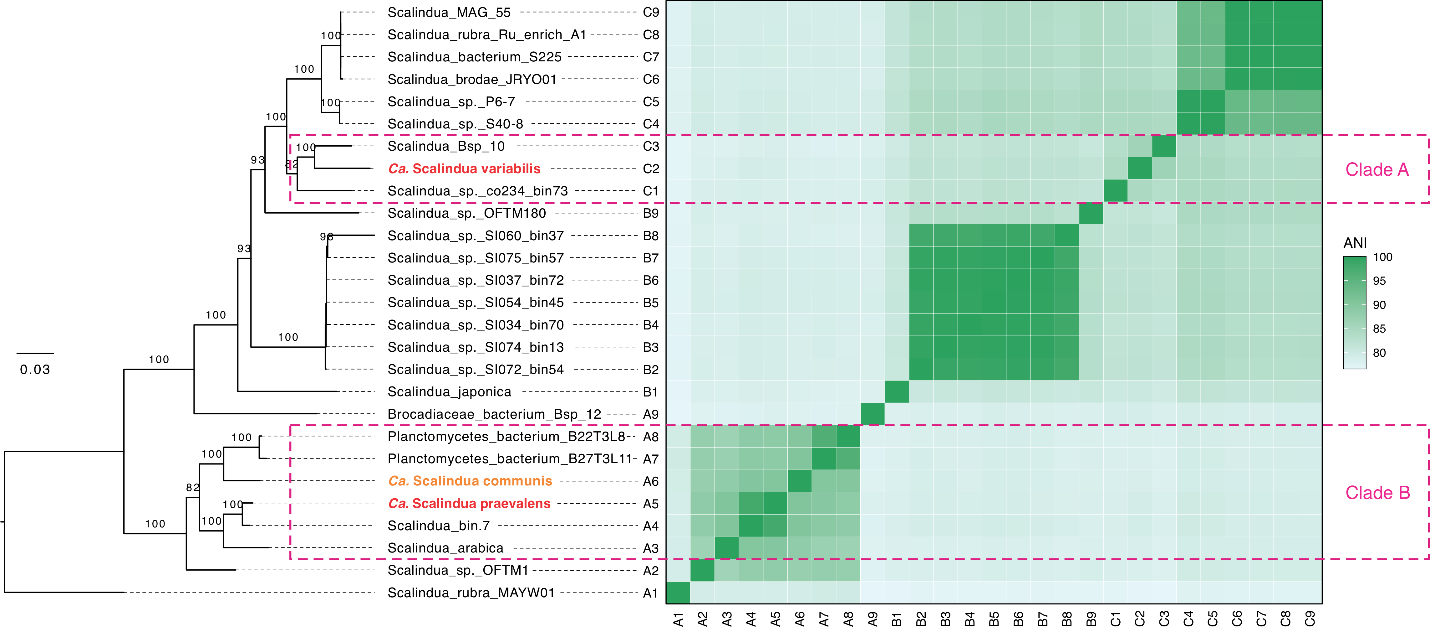


**Fig. S2. Average nucleotide identities (ANIs) between *Scalindua* genomes.** The metagenome-assembled genomes (MAGs) recovered from the Arabian Sea ODZ are highlighted in red, while that from AMOR sediments is shown in orange. The maximum-likelihood phylogenetic tree on the left is based on 120 bacterial single-copy genes. The two clades containing the two ODZ *Scalindua* bacteria are highlighted by dashed rectangle boxes.

**
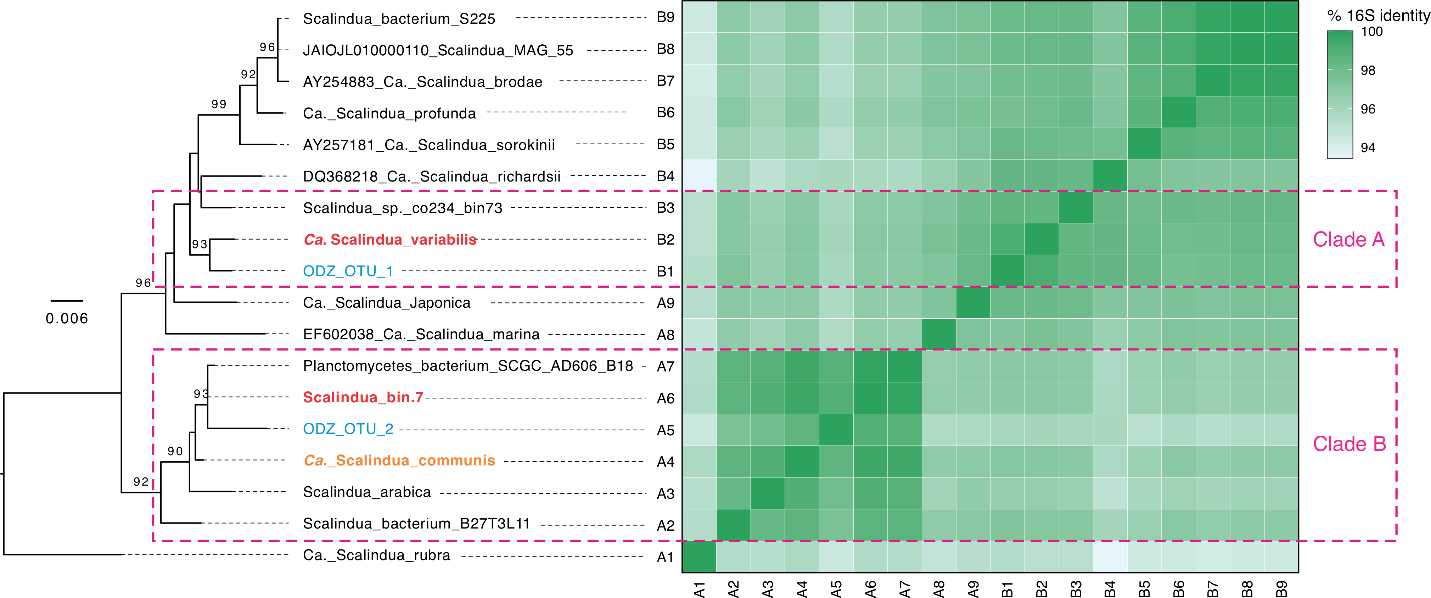
**

**Fig. S3. Pair-wise 16S rRNA gene identities between *Scalindua* genomes.** The metagenome-assembled genomes (MAGs) recovered from ODZs are highlighted in red, while that from AMOR sediments is shown in orange. The maximum-likelihood phylogenetic tree on the left is based on 120 bacterial single-copy genes. The two clades containing the two ODZ *Scalindua* bacteria are highlighted by dashed rectangle boxes.

**
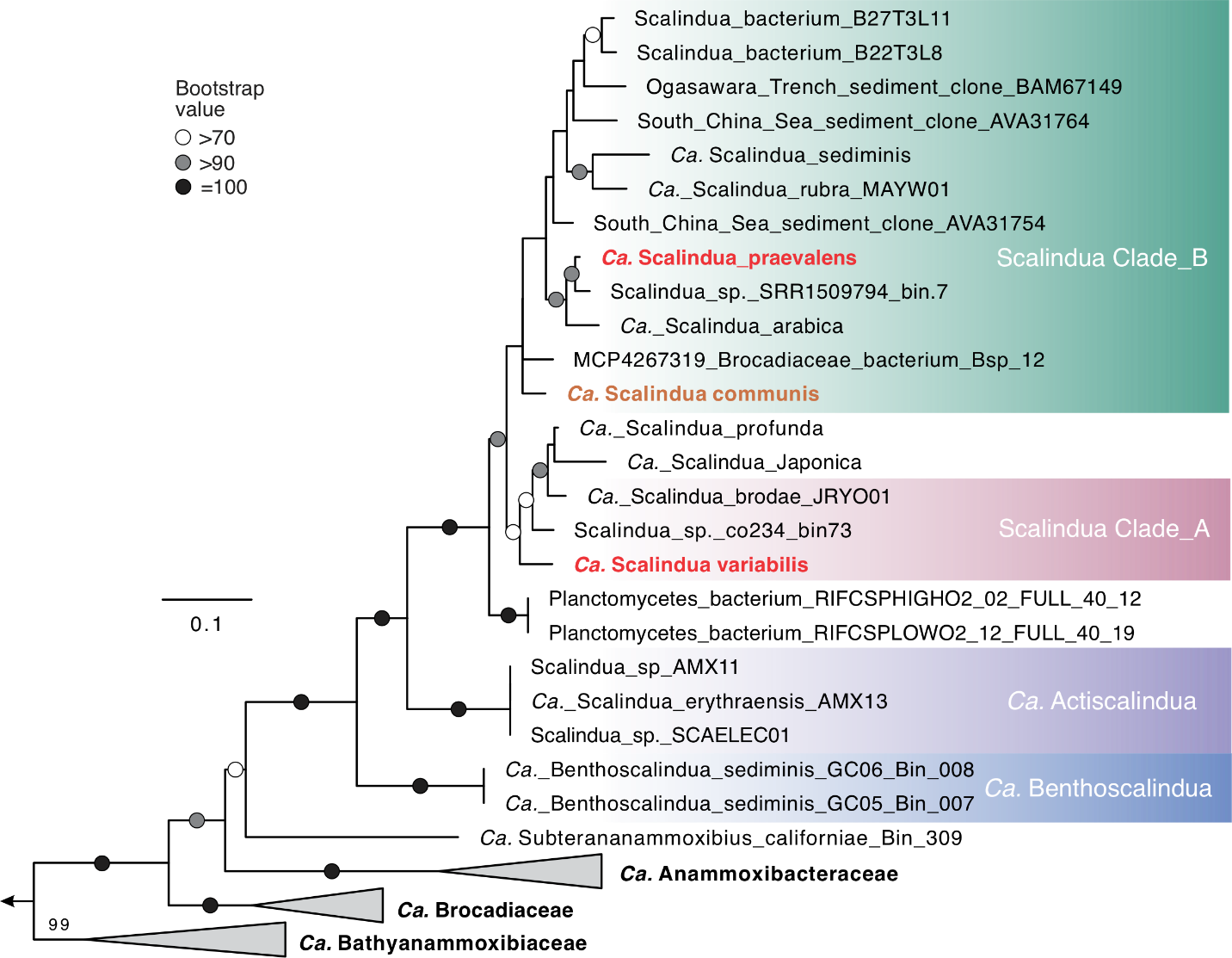
**

**Fig. S4. Maximum-likelihood phylogenetic tree of the hydrazine synthase alpha subunit (HzsA) of anammox bacteria.** For simplicity, only lineages within the *Ca.* Scalinduaceae family are shown, while other families are collapsed. The two ODZ MAGs are highlighted in red, while the sediment MAG is shown in orange. Bootstrap values of >70 (*n* = 1000) are shown with symbols listed in the legend. The scale bar shows estimated sequence substitutions per residue.

**
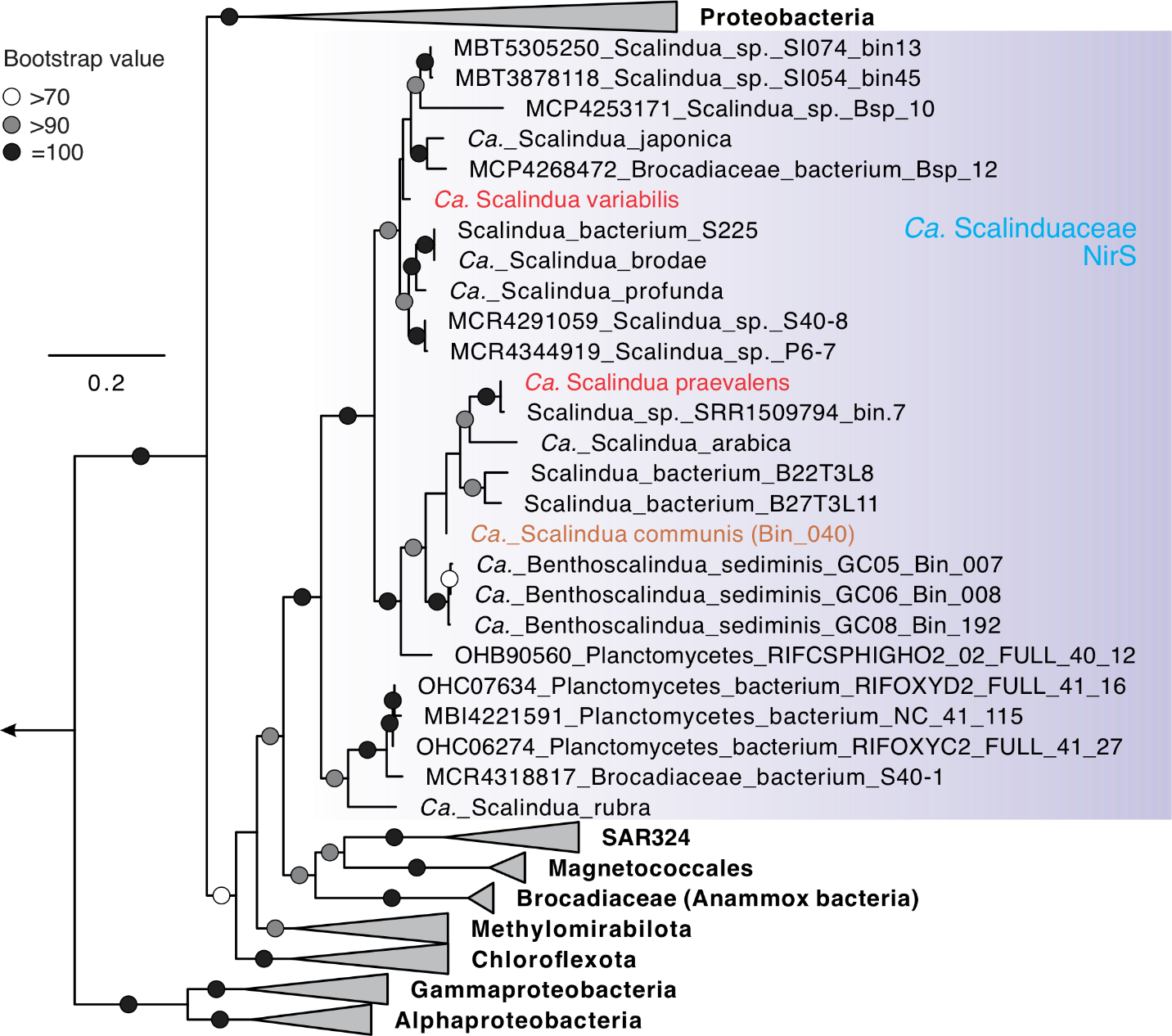
**

**Fig. S5. Maximum-likelihood phylogenetic tree of cytochrome *cd1* nitrite reductase (NirS) in anammox bacteria.** For simplicity, only the clade of anammox bacteria is shown, while other clades are collapsed. The two ODZ anammox MAGs are highlighted in red, while the sediment MAG is shown in orange. Bootstrap values of >70 (*n* = 1000) are shown with symbols listed in the legend. The scale bar shows estimated sequence substitutions per residue.

**
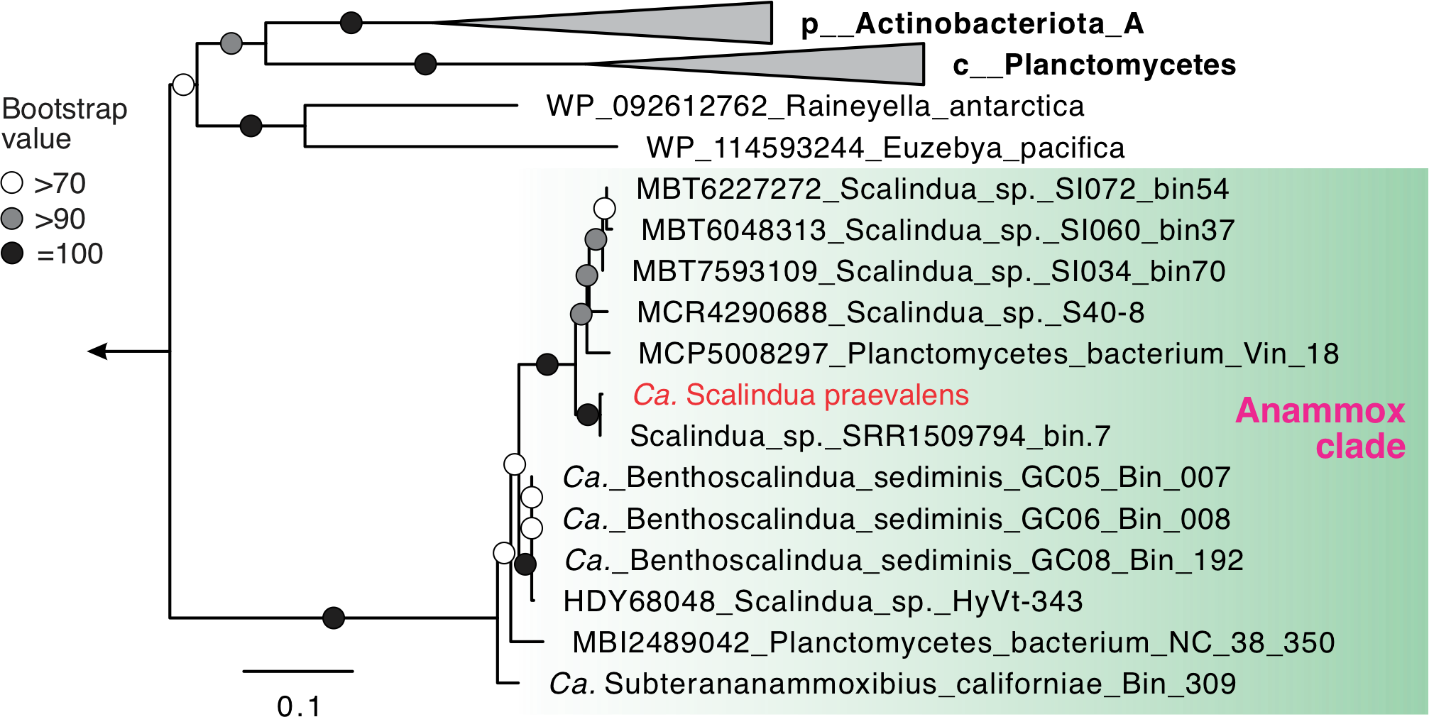
**

**Fig. S6. Maximum-likelihood phylogenetic tree of urease alpha subunit (UreC) in anammox bacteria.** For simplicity, only the clade of the marine anammox clade is shown, while other clades are collapsed if possible. The urease-containing ODZ anammox bacterium is highlighted in red. Bootstrap values of >70 (*n* = 1000) are shown with symbols listed in the legend. The scale bar shows estimated sequence substitutions per residue.

**
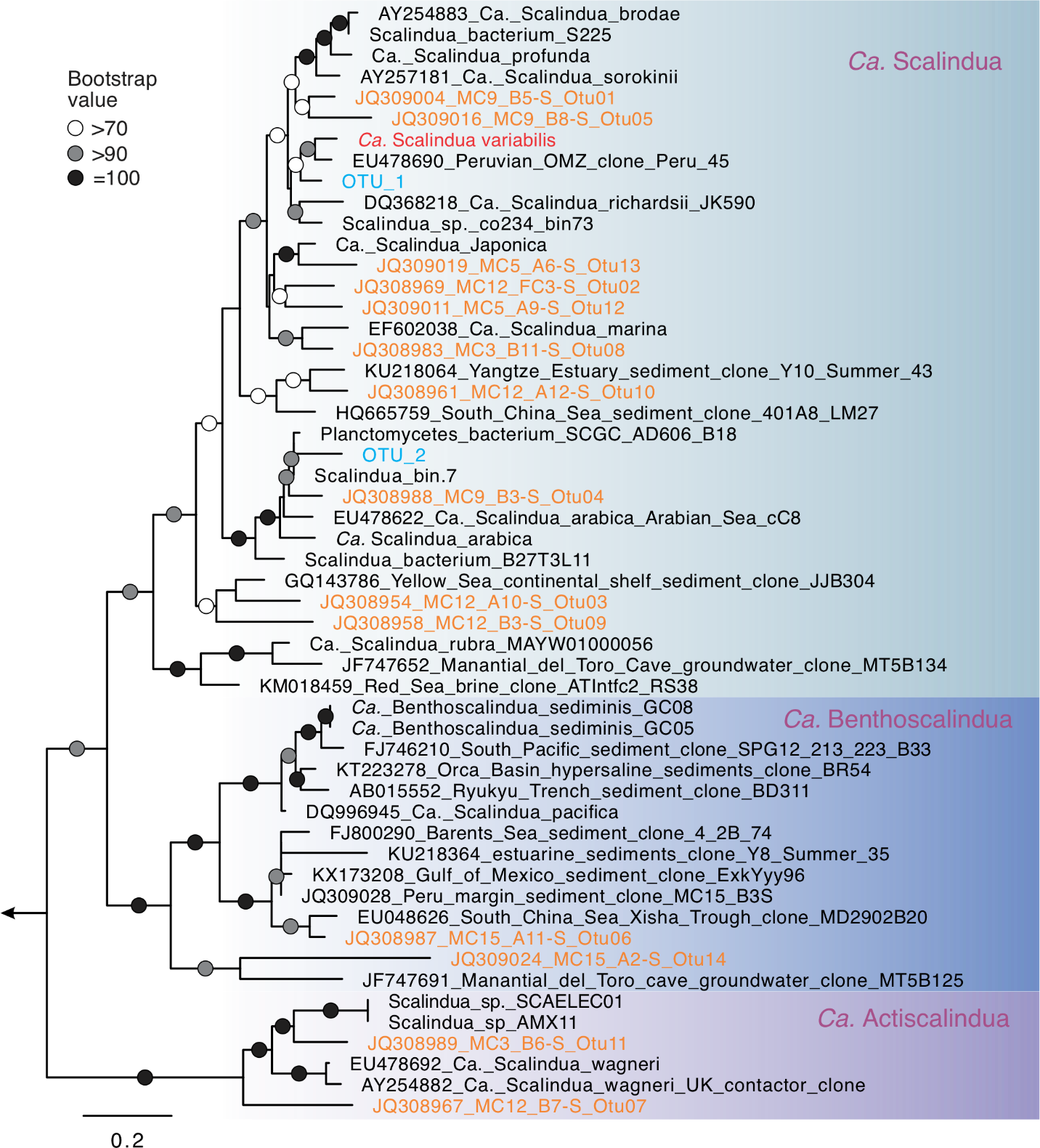
**

**Fig. S7. Phylogenetic affiliations of diverse *Scalindua* bacteria in sediments beneath the Peru margin oxygen deficient zone.** The phylogenetic tree is inferred based on the 16S rRNA gene sequences reported in [7]. The sediment *Scalindua* is distributed in all three known genera in the *Ca.* Scalinduaceae family. The 14 OTU sequences from the Peru margin sediments are highlighted in orange, while the two from global ODZs are shown in blue. *Ca.* Scalindua variabilis from ODZ is shown in red. Bootstrap values of >70 (*n* = 1000) are shown with symbols listed in the legend. The scale bar shows estimated sequence substitutions per residue.


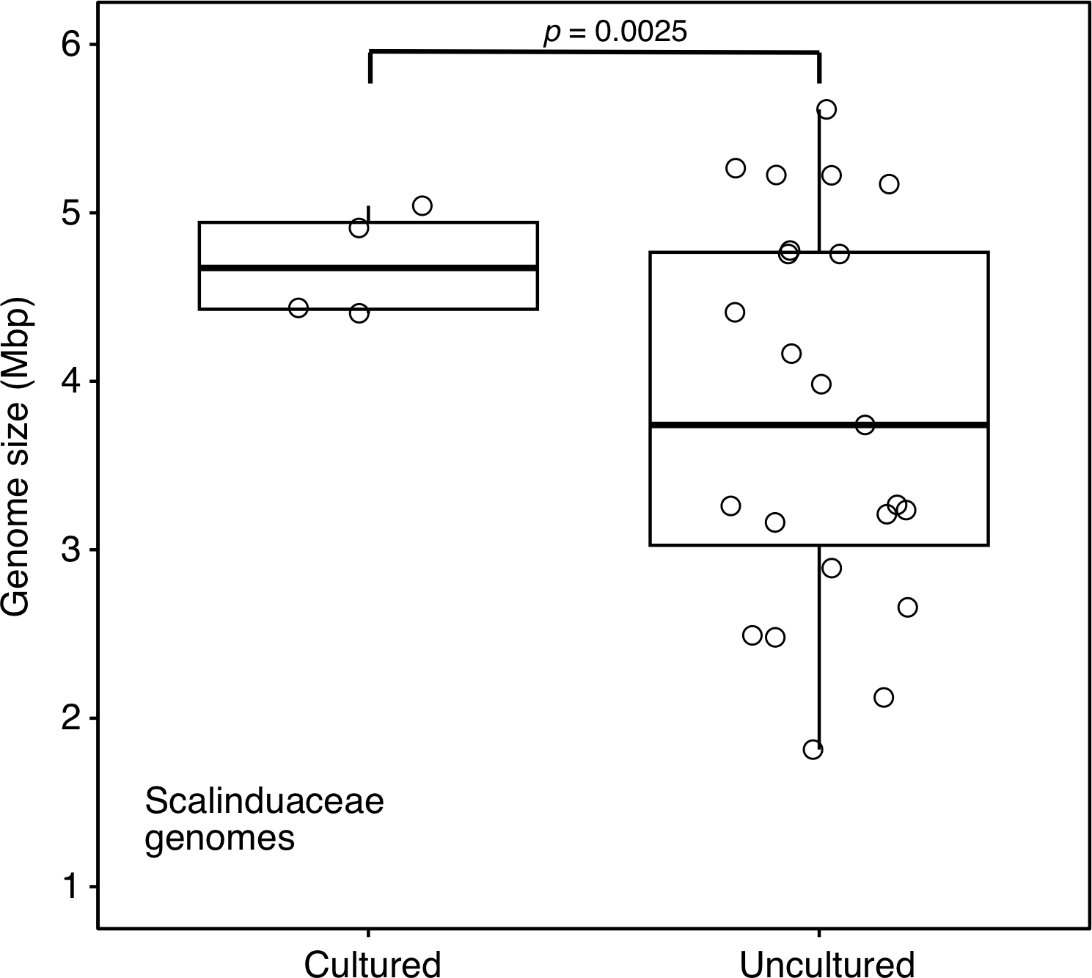


**Fig. S8. Genome size comparison between the cultured and uncultured anammox bacteria affiliated with the *Ca.* Scalinduaceae family.** In the boxplot, the boxes contain values between the first and third quartiles, while the thick horizontal lines indicate the median values. The genome sizes of anammox bacteria are shown in open circles.

**Supplementary Tables**

**Table S1. Anammox bacterial 16S rRNA gene sequences in ODZ metagenome assemblies**

| Location | Metagenome SRA/JGI ID | Depth* (m) | # Brocadiales 16S rRNA  gene sequence | 16S rRNA gene phylogenetic affiliation |
| --- | --- | --- | --- | --- |
| Arabian Sea | AMALJGI-DNA-9 | 130 | 1 | *Ca.* S. variabilis |
|  | AMALJGI-DNA-10 | 150 | 1 | *Ca.* S. praevalens |
|  | AMALJGI-DNA-11 | 200 | 1 | *Ca.* S. praevalens |
|  | AMALJGI-DNA-12 | 400 | 1 | *Ca.* S. praevalens |
|  | Arabian Sea co-assembly [8] | -- | 1 | *Ca.* S. praevalens |
| ETNP | AMALJGI-DNA-1 | 53 | 0 | -- |
|  | AMALJGI-DNA-2 | 120 | 1 | *Ca.* S. praevalens |
|  | AMALJGI-DNA-3 | 200 | 1 | *Ca.* S. praevalens |
|  | AMALJGI-DNA-5 | 10 | 0 |  |
|  | AMALJGI-DNA-7 | 185 | 1 | *Ca.* S. praevalens |
|  | AMALJGI-DNA-8 | 215 | 1 | *Ca.* S. praevalens |
|  | AMALJGI-DNA-13 | 60 | 0 | -- |
|  | AMALJGI-DNA-14 | 95 | 0 | -- |
|  | AMALJGI-DNA-15 | 200 | 0 | -- |
|  | AMALJGI-DNA-17 | 16 | 0 | -- |
|  | AMALJGI-DNA-18 | 45 | 0 | -- |
|  | AMALJGI-DNA-20 | 250 | 1 | *Ca.* S. praevalens |
|  | Fuchsman co-assembly [9] | -- | 1 | *Ca.* S. praevalens |
|  | Glass co-assembly [10] | -- | 1 | *Ca.* S. praevalens |

*ODZ depths are highlighted in blue, while co-assemblies are shown in yellow. --, not relevant.

**Table S2 Matches between the ODZ *Scalindua* MAGs and the dominant OTUs in Peru Margin sediments**

| Genomes | Peru sediment OTUs | 16S rRNA  gene identity | Belong to the  same species? |
| --- | --- | --- | --- |
| *Scalindua* ODZ_A | Peru_OTU_1  (JQ309004_MC9_B5-S_Otu1 in Fig. S6) | 98.6% | Yes |
| *Scalindua* ODZ_B | Peru_OTU_4  (JQ308988_MC9_B3-S_Otu4 in Fig. S6) | 98.6% | Yes |

**Supplementary References**

1. Zhao, R., et al., Geochemical transition zone powering microbial growth in subsurface sediments. Proceedings of the National Academy of Sciences. 2020;117:32617-32626.

2. Shaw, D.R., et al., Extracellular electron transfer-dependent anaerobic oxidation of ammonium by anammox bacteria. Nature Communications. 2020;11:2058.

3. Ali, M., D.R. Shaw, and P.E. Saikaly, Application of an enrichment culture of the marine anammox bacterium “Ca. Scalindua sp. AMX11” for nitrogen removal under moderate salinity and in the presence of organic carbon. Water Research. 2020;170:115345.

4. Schauberger, C., et al., Microbial community structure in hadal sediments: high similarity along trench axes and strong changes along redox gradients. The ISME Journal. 2021;15:3455-3467.

5. Zhao, R., S.L.M. Bauer, and A.R. Babbin, "Candidatus Subterrananammoxibiaceae", a new anammox bacterial family in globally distributed marine and terrestrial subsurfaces. Applied and environmental microbiology. 2023;89:e00800-23.

6. Thamdrup, B., et al., Anammox bacteria drive fixed nitrogen loss in hadal trench sediments. Proceedings of the National Academy of Sciences. 2021;118:e2104529118.

7. Rich, J.J., et al., Anaerobic ammonium oxidation (anammox) and denitrification in Peru margin sediments. Journal of Marine Systems. 2020;207:103122.

8. Zhang, I.H., et al., Partitioning of the denitrification pathway and other nitrite metabolisms within global oxygen deficient zones. ISME Communications. 2023;3:76.

9. Fuchsman, C.A., et al., Niche partitioning of the N cycling microbial community of an offshore oxygen deficient zone. Frontiers in Microbiology. 2017;8:2384.

10. Glass, J.B., et al., Meta-omic signatures of microbial metal and nitrogen cycling in marine oxygen minimum zones. Frontiers in Microbiology. 2015;6:998.
